# Transcriptome Analysis of Ullrich Congenital Muscular Dystrophy Fibroblasts Reveals a Disease Extracellular Matrix Signature and Key Molecular Regulators

**DOI:** 10.1371/journal.pone.0145107

**Published:** 2015-12-15

**Authors:** Sonia Paco, Teresa Casserras, Maria Angels Rodríguez, Cristina Jou, Montserrat Puigdelloses, Carlos I. Ortez, Jordi Diaz-Manera, Eduardo Gallardo, Jaume Colomer, Andrés Nascimento, Susana G. Kalko, Cecilia Jimenez-Mallebrera

**Affiliations:** 1 Neuromuscular Unit, Neuropaediatrics Department, Hospital Sant Joan de Déu, Fundación Sant Joan de Déu, Barcelona, Spain; 2 Bioinformatics Core Facility, IDIBAPS, Hospital Clinic, Barcelona, Spain; 3 Pathology Department, Hospital Sant Joan de Déu, Barcelona, Spain; 4 Neuromuscular Diseases Unit, Neurology Department, Hospital de la Santa Creu i Sant Pau, Universidad Autónoma de Barcelona, Barcelona, Spain; 5 Center for Biomedical Research on Rare Diseases (CIBERER), Instituto de Salud Carlos III, Madrid, Spain; University of Valencia, SPAIN

## Abstract

**Background:**

Collagen VI related myopathies encompass a range of phenotypes with involvement of skeletal muscle, skin and other connective tissues. They represent a severe and relatively common form of congenital disease for which there is no treatment. Collagen VI in skeletal muscle and skin is produced by fibroblasts.

**Aims & Methods:**

In order to gain insight into the consequences of collagen VI mutations and identify key disease pathways we performed global gene expression analysis of dermal fibroblasts from patients with Ullrich Congenital Muscular Dystrophy with and without vitamin C treatment. The expression data were integrated using a range of systems biology tools. Results were validated by real-time PCR, western blotting and functional assays.

**Findings:**

We found significant changes in the expression levels of almost 600 genes between collagen VI deficient and control fibroblasts. Highly regulated genes included extracellular matrix components and surface receptors, including integrins, indicating a shift in the interaction between the cell and its environment. This was accompanied by a significant increase in fibroblasts adhesion to laminin. The observed changes in gene expression profiling may be under the control of two miRNAs, miR-30c and miR-181a, which we found elevated in tissue and serum from patients and which could represent novel biomarkers for muscular dystrophy. Finally, the response to vitamin C of collagen VI mutated fibroblasts significantly differed from healthy fibroblasts. Vitamin C treatment was able to revert the expression of some key genes to levels found in control cells raising the possibility of a beneficial effect of vitamin C as a modulator of some of the pathological aspects of collagen VI related diseases.

## Introduction

Ullrich congenital muscular dystrophy (UCMD) is caused by mutations in collagen VI genes (*COL6A1*, *COL6A2 and COL6A3*) and is characterised by congenital hypotonia, proximal muscle weakness and distal joint hyperlaxity. Multiple joint contractures and progressive respiratory insufficiency develop over time [1,2]. In addition to these skeletal features, UCMD patients show a recognisable skin pathology in the form of follicular hyperkeratosis pilaris and abnormal scarring following skin injury mainly in the form of hypertrophic scars and keloids [3,4]. These constitute a fibrotic process due to the excessive deposition of collagen and other extracellular matrix proteins during the wound healing process [5] and can cause pain and pruritus and significant discomfort in patients that require frequent surgical interventions. Expression of collagen VI mRNA has been shown to increase in the early phases of wound healing and in keloids [6] as well as in other fibrotic processes such as systemic sclerosis [7] suggesting a role in matrix reorganisation, fibrosis and scaring in general. Wound healing of tendons and ligaments is generally similar to that of skin [8]. Collagen VI is abundant in the extracellular matrix of many tissues, where it binds to cell-surface receptors, integrins [9] and NG2 [10] and to other extracellular matrix (ECM) components including fibrillar collagens, collagen IV and fibronectin. Via these interactions collagen VI mediates cell adhesion and extracellular matrix organisation [11–13]. Collagen also VI triggers intracellular signaling events regulating for instance cell cycle progression [14] and apoptosis [15].

Fibroblasts, which are the main source of collagen VI, also synthesise and secrete many other ECM proteins, growth factors, matrix metalloproteinases and other soluble molecules such as chemokines which are necessary to maintain correct tissue homeostasis and function [16].

In order to investigate the effect of collagen VI mutations on skin fibroblasts function and how they may contribute to abnormal scarring and other pathological features seen in collagen VI deficiency we performed global gene expression profiling of skin fibroblasts from healthy controls and UCMD patients. Albeit the recognized differences between skin, muscle and other tissue specific fibroblasts we thought that this approach may also help us shed some light into the mechanisms controlling some of the skeletal changes that are observed in patients with collagen VI defects.

Vitamin C is necessary for the hydroxylation of lysyl residues and secretion of different collagen types. In addition, it is a potent antioxidant and has other cellular functions such as promoting cell proliferation, migration, and regulation of gene expression [17]. To find out if collagen VI deficiency has an effect on the fibroblast response to vitamin C we also compared UCMD and healthy fibroblasts that had been previously treated with ascorbic acid.

## Materials and Methods

### Ethics Statement

This work has been approved by the Ethical Committee of “Fundació Sant Joan de Déu”. Written informed consent for research was obtained from all patients and controls (or their parents/guardians) according to the Hospital Sant Joan de Déu forms and regulations.

### Patients and samples

UCMD patients with confirmed mutations in *COL6A* genes have been described previously [18]. All patients showed a deficiency in collagen VI secretion by dermal fibroblasts that ranged from a mild to a severe reduction in extracellular collagen VI.

Skin biopsies from the forearm were obtained from UCMD and Bethlem myopathy (BM) patients and from children not affected by a neuromuscular condition. In the microarray analysis we included 6 UCMD and 6 aged-matched control fibroblast cell lines Primary skin fibroblasts cultures were established as previously described [19]. Confluent fibroblasts (passages 2 or 3) were treated or not with 50 μg/mL of L-ascorbic acid phosphate magnesium (Wako Chemicals GmbH, Neuss, Germany) for 5 days before RNA extraction. Open quadriceps muscle biopsies were snap-frozen in liquid nitrogen before RNA extraction. Serum samples were collected, separated by centrifugation and stored at -80°C until the moment of the analysis.

### RNA isolation

Total RNA was extracted with RNeasy Fibrous Tissue mini kit (Qiagen, Hilden, Germany) and its quantity and quality assessed as previously described [18]. For miRNA isolation from serum Trizol extraction was applied.

### Microarray and Systems Biology Analysis

Expression data have been submitted to NCBI’s Gene Expression Omnibus database (GSE56741, http://www.ncbi.nlm.nih.gov/geo/query/acc.cgi?acc=GSE56741).

Biotin-labelled and fragmented target RNA samples were loaded into Affymetrix GeneChip^®^ (Human Genome U219) Array Plate (Affymetrix, Santa Clara, CA, USA). The current format of these arrays interrogates more than 36,000 transcripts and variants, which represent more than 20,000 annotated genes in the human genome. Washes and scanning of the arrays were performed according to manufacturer instructions. Raw data was normalized using Robust Multichip Analysis (RMA) method to assure comparability across samples [20]. Statistical differential gene expression analysis between groups was made by the non-parametric approach Rank Prod [21] which detects genes that are consistently highly ranked in a number of replicate experiments. Those Affymetrix probesets having changes between groups with false discovery rate (FDR) lower than 0.05 were considered significant. David tool [22] was used for the functional enrichment analysis using significant gene lists, and Gene Ontology Biological Process (www.geneontology.org) and KEGG pathways (Kyoto Encyclopedia of Genes and Genomes, www.genome.jp/kegg) databases were considered.

Physical interaction networks have been constructed using the Ingenuity Pathways tool (IPA) (www.ingenuity.com), based on extensive records maintained in the Ingenuity Pathways Knowledge Base (IPKB) database.

Pearson correlations were computed considering expression values of interesting genes to construct and display networks using Cytoscape tool (|R|>0.8, p-value<0.005) [23].

### Real-Time Quantitative RT-PCR

High-throughput real-time qPCR was performed on the BioMark 48.48 Dynamic Array (Fluidigm^®^, South San Francisco, CA, USA) with Taqman Gene Expression Assays as previously described [24]. *HPRT1* was used as the housekeeping gene.

### Adhesion assay

Fibroblasts were seeded into 96-well culture 8 well-strips coated with vitronectin, fibronectin, laminin, collagen type I and collagen type IV (Millicoat^™^ Screening Kit, Millipore, Billerica, MA, USA). Cells were incubated for 1 hour at 37°C/ 5% CO_2_. The adherent cells were washed with HBSS and stained with 0.2% crystal violet solution in 10% ethanol for 5 min. Excess dye was removed by washing with PBS, the violet stain was solubilized with a mixture of 0.1M NaH_2_PO_4_ pH 4.5 and 50% ethanol and absorbance was measured at 540 nm on a microplate reader (Emax, Molecular Devices).

### Immunofluorescence and Western blot

Fibroblasts were fixed in 2% paraformaldehyde on ice for 10 minutes. Blocking was performed using 2% BSA in PBS-Tween 0.05%. Mouse monoclonal anti-integrin- α3 (Millipore MAB1952, 1:50) was used and detected with a secondary anti-mouse IgG conjugated with Alexa-488. Images were captured with a Leica epifluorecent microscope and LAS software. For Western blot analysis total protein extracts were separated on 10% Mini-PROTEAN ^®^ TGX (Bio-Rad, Hercules, CA, USA) and transferred onto a nitrocellulose membrane using Trans-Blot^®^ TurboTM Mini Nitrocellulose Transfer Pack and Tans-Blot^®^ Turbo TM Transfer System (BioRad, Richmond, USA). After blocking, membranes were incubated with primary antibodies over night at 4°C. A HRP-conjugated donkey anti -mouse secondary antibody (Jackson Immuno Research) was used. Proteins were detected using chemiluminescence (ECL, Pierce, Rockford, USA). The protein content was expressed in arbitrary units relative to α-Tubulin (Sigma, Missouri, USA) as the protein loading control. The intensity of protein bands was determined by densitometry with ImageJ Software.

### miRNA analysis

miRNA analysis was performed as described previously [25]. Real-time PCR was performed in duplicates using Taqman Gene Expression Master Mix and Taqman Assays for selected miRNAs (U6 snRNA, hsa-miR-181a and hsa-miR-30c) in 7500 real-time PCR System using Applied Biosystems software v.2.0.4 (Foster City, CA, USA). U6 snRNA was used as a reference to normalize transcription levels among patients. Fold changes were calculated as mean values of 2-ΔΔCT or 1/2-ΔΔCT relative to control. A fold change above or below 1.5 was considered significant.

## Results

### Overview

Statistical analysis with Rank Prod revealed important changes in gene expression in all four comparisons analysed (Table 1). The complete list of unique under-expressed and over-expressed genes in each comparison is provided in S1 Table. The Top 10 down- and up-regulated genes are summarised in Table 2 for P-C comparison (untreated patient versus untreated control cells) and in S2 Table for the other three comparisons. These changes were validated for a selection of genes of interest by real-time PCR (S3 Table). There were 314 genes in common (approximately 50% for both comparisons) between P-C and PAA-CAA comparisons, mainly enriching TGF-beta signaling and ECM-receptor interaction pathways (see below). We recently described the transcriptomic profile of UCMD muscle [24]. We compared those data with the results from the fibroblasts cultures and found 52 differentially expressed genes in common. KEGG pathway analysis of those common genes revealed that they were involved either in immunity or cell adhesion (Table 3). In the first group we found several genes for class I and class II antigens as well as components of the complement cascade indicating that collagen VI deficiency leads to an inflammatory signal both in skin and muscle. Regarding cell adhesion we found similar fold changes in fibroblasts and muscle in common genes including those for laminin-α4, tenascin X, caveolin-1, thrombospondin-4 and WNT1 inducible signaling pathway protein (WISP2). Thus, these proteins may play an important role in disease pathogenesis.

**Table 1:** Differentially expressed genes in each contrast (FDR<0.05).

Unique genes/Contrast	P-C	P_AA_-P	P_AA_-C_AA_	C_AA_-C
UP	360	298	442	540
DOWN	233	276	189	544
TOTAL	593	575	631	1084

P: patients; C: controls; P_AA_: patients treated with ascorbic acid (AA); C_AA_: controls treated with AA. FDR: false discovery rate.

**Table 2:** Top-ten down- and up-regulated genes in the comparison P-C.

Gene symbol	Gene name	FC	FDR
*DACT1*	dishevelled-binding antagonist of beta-catenin 1	-6.31	0
*SCRG1*	stimulator of chondrogenesis 1	-5.88	0
*ID4*	inhibitor of DNA binding 4, dominant negative helix-loop-helix protein	-4.31	0
*COL11A1*	collagen, type XI, alpha 1	-4.01	0
*GPC4*	glypican 4	-3.94	0
*RDH10*	retinol dehydrogenase 10 (all-trans)	-3.58	0
*TPD52L1*	tumor protein D52-like 1	-3.57	0
*GDF6*	growth differentiation factor 6	-3.47	0
*INHBE*	inhibin, beta E	-3.30	0
*SLC38A4*	solute carrier family 38, member 4	-3.15	0
*APCDD1*	adenomatosis polyposis coli down-regulated 1	10.34	0
*C10orf116*	adipogenesis regulatory factor (ADIRF)	9.06	0
*EPDR1*	ependymin related 1	5.09	0
*CLEC3B*	C-type lectin domain family 3, member B	5.01	0
*INHBB*	inhibin, beta B	4.91	0
*LY6K*	lymphocyte antigen 6 complex, locus K	4.90	0
*OLFML2A*	olfactomedin-like 2A	4.73	0
*NTN4*	netrin 4	4.67	0
*SLC7A14*	solute carrier family 7, member 14	4.41	0
*PSPH*	phosphoserine phosphatase	4.23	0

**Table 3:** KEGG pathway analysis of genes commonly regulated in collagen VI deficient skeletalmuscle (Paco et al., 2103) and skin fibroblasts (FDR<30).

KEGG Pathway	Genes	FDR
Type I diabetes mellitus	*IGF2*, *HLA-DPA1*, *HLA-DPB1*, *HLA-F*	6.28E-04
Viral myocarditis	*CAV1*, *HLA-DPA1*, *HLA-DPB1*, *HLA-F*	0.003
Focal adhesion	*CAV1*, *LAMA4*, *IGF1*, *COL1A1*, *THBS4*	0.008
Allograft rejection	*HLA-DPA1*, *HLA-DPB1*, *HLA-F*	0.009
Graft-versus-host disease	*HLA-DPA1*, *HLA-DPB1*, *HLA-F*	0.01
Cell adhesion molecules (CAMs)	*HLA-DPA1*, *HLA-DPB1*, *JAM2*, *HLA-F*	0.016
Autoimmune thyroid disease	*HLA-DPA1*, *HLA-DPB1*, *HLA-F*	0.018
Complement and coagulation cascades	*F10*, *C3*, *CFD*	0.032

### Functional Enrichment analysis:

#### Patient versus control fibroblasts

Gene Ontology (GO) and KEGG pathway databases were used for functional enrichment analysis as previously described [24]. The most over-represented GO_BP terms amongst up-regulated genes were related to cell adhesion, immunity, blood vessel development and wound healing (S4 Table). Hypertrophic scars and keloids, which are common in collagen VI deficiency, are a manifestation of abnormal wound healing. Wound healing is a highly orchestrated process which consists of a series of stages: hemostasis and coagulation, inflammation, proliferation and remodeling [8], [5]. Within the GO terms related to scaring and blood vessel development we found genes involved in one or more stages of wound healing as summarized in Table 4.

**Table 4:** Genes involved in wound healing that are either up-regulated or down-regulated in P-C fibroblasts comparison.

*Coagulation*	*Inflammation*	*Angiogenesis*	*Adhesion*	*ECM remodelling*
*F10* (+2.6) coagulation factor X	*CD59* (+1.8) inhibitor of complement membrane attack	*COL18A1* (+.2.2) precursor of endostatin	*CD44* (+1.8), *ITGA7* (+2.8)	*TIMP3* (+1.7) inhibitor of matrix metalloproteinases
*SERPINB2* (+2.4) *TFPi* (+2.2) Protease inhibitors	*CD40* (+1.7) IFN-γ and CD40L receptor	*MEOX2* (+2.2), *ITGA7* (+2.8), vascular smooth muscle development	*CD9* (+3.8)	*PLAUR* (+1.8) receptor for urokinase plasminogen activator
*PLAUR* (+1.8) receptor for urokinase plasminogen activator	*C3* (-2.2), *CFD* (-1.6) complement cascade	*TBX1* (+2.7), *ANPEP* (+1.9), *ARHGAP22* (+1.9), *CD44* (+1.8) Endothelial cell formation and proliferation		
	*HLA-DPA1* (+4), *HLA-DPB1* (+2.2), *HLA-F* (+1.8), major histocompatibility complex II and I	*LAMA4* (+ 1.7), *ENG* (+1.8)) endothelial cell membrane and basal lamina		
	*SERPING1* (+1.7), complement 1 inhibitor			

On the other hand, KEGG database enrichment analysis identified 9 significantly enriched pathways for the total 593 differentiated genes in untreated patient cells relative to untreated control cells. The top 3 pathways were ECM-receptor interaction, nitrogen metabolism and TGF-β signaling (Table 5). Nitrogen metabolism genes included cysteine-protein-sulfhydrase (*CTH* -2.5), which is involved in the synthesis of cysteine and asparagine synthetase (*ASNS* -2.11) which is induced by starvation. Glutamate-ammonia ligase (*GLUL* +1.7) is responsible for the synthesis of glutamine from glutamate and ammonia whereas the enzyme that catalyses the reverse reaction in mitochondria was under-expressed (*GLS* -2.21). Both enzymes regulate ammonia levels and therefore acid-base balance. Also, in this category we found changes in cytosolic and extracellular isoforms of carbonic anhydrase (*CA13*–1.65 and *CA12* +2.5) which are necessary for maintaining tissue PH. These data suggest that collagen VI deficiency in fibroblasts may result in imbalances of PH and an adaptative metabolic response involving amino acids.

**Table 5:** KEGG pathway analysis of all regulated genes in P-C comparison with an FDR ≤ 0.3.

KEGG Pathway	*Genes*	FDR
ECM-receptor interaction	*COL4A1*, *TNXB*, *TNXA*, *TNC*, *ITGA3*, *LAMA4*, *ITGA6*, *CD44*, *COMP*, *ITGA7*, *COL1A1*, *COL11A1*, *THBS4*	5.30E-04
Nitrogen metabolism	*CTH*, *GLUL*, *CA13*, *CA12*, *GLS*, *ASNS*	0.002
TGF-beta signaling pathway	*INHBB*, *ACVR2A*, *INHBA*, *INHBE*, *COMP*, *GDF6*, *GDF5*, *ID4*, *DCN*, *ID3*, *THBS4*	0.002
Arrhythmogenic right ventricular cardiomyopathy (ARVC)	*JUP*, *TCF7*, *DSG2*, *SGCG*, *ITGA6*, *ITGA7*, *LMNA*, *SGCD*, *ITGA3*, *TCF7L2*	0.003
Complement and coagulation cascades	*F10*, *MASP1*, *C3*, *F3*, *CD59*, *TFPI*, *SERPING1*, *CFD*, *PLAUR*	0.006
Hypertrophic cardiomyopathy (HCM)	*ACTC1*, *IL6*, *SGCG*, *ITGA6*, *ITGA7*, *LMNA*, *IGF1*, *SGCD*, *ITGA3*, *TPM1*	0.007
Viral myocarditis	*CAV1*, *SGCG*, *MYH2*, *SGCD*, *HLA-DPA1*, *HLA-DPB1*, *CD40*, *HLA-DRA*, *HLA-F*	0.008
Hematopoietic cell lineage	*CD9*, *IL6*, *CD44*, *ITGA6*, *CD59*, *ANPEP*, *ITGA3*, *CD14*, *HLA-DRA*	0.023
Renin-angiotensin system	*AGTR1*, *AGT*, *ANPEP*, *ENPEP*	0.03

It has been previously described that TGF-β signaling contributes to muscular dystrophies and myopathies by promoting fibrosis formation [26]. Expression of different components of this pathway including members of TGF-β superfamily of cytokines such as inhibins (*INHBA -1*.*96*, *INHBE -3*.*3* and *INHBB*, +4.90), growth differentiation factors (*GDF5* +2.16 and *GDF6–3*.*47*), ECM components that modulate the ligand access to TGF-β receptors (*COMP +1*.*68*, *DCN* +1.82 and *THBS4 +1*.*85*), type II transmembrane serine/threonine kinase receptor (*ACVR2A -1*.*76*) and effectors of down-stream Smad signaling pathway (*ID3–1*.*74* and *ID4–4*.*31*) were significantly either up-regulated or down-regulated in our dataset. The TGF-beta signaling pathway is complex and while some of those components may be exerting a stimulatory effect on fibrosis others may be acting on the opposite direction and therefore it is difficult to foresee from changes at gene expression level whether this pathway is activated or not. We studied phosphorylation of Smad2 after treatment with TGF-β of UCMD and control cells and although we detected phosphorylation of Smad2 we did not find significant differences between patients and control cells (data not shown). In addition, IPA tool could not find statistical evidence of this pathway being either activated or inhibited.

#### ECM-receptor interaction and cell adhesion

Extracellular Matrix (ECM)—receptor was the most highly over-represented KEGG canonical pathway with 12 genes (Table 5). Given the position of collagen VI as a key component of the ECM we decided to focus on this pathway for a more detailed analysis.

**Fig 1:**
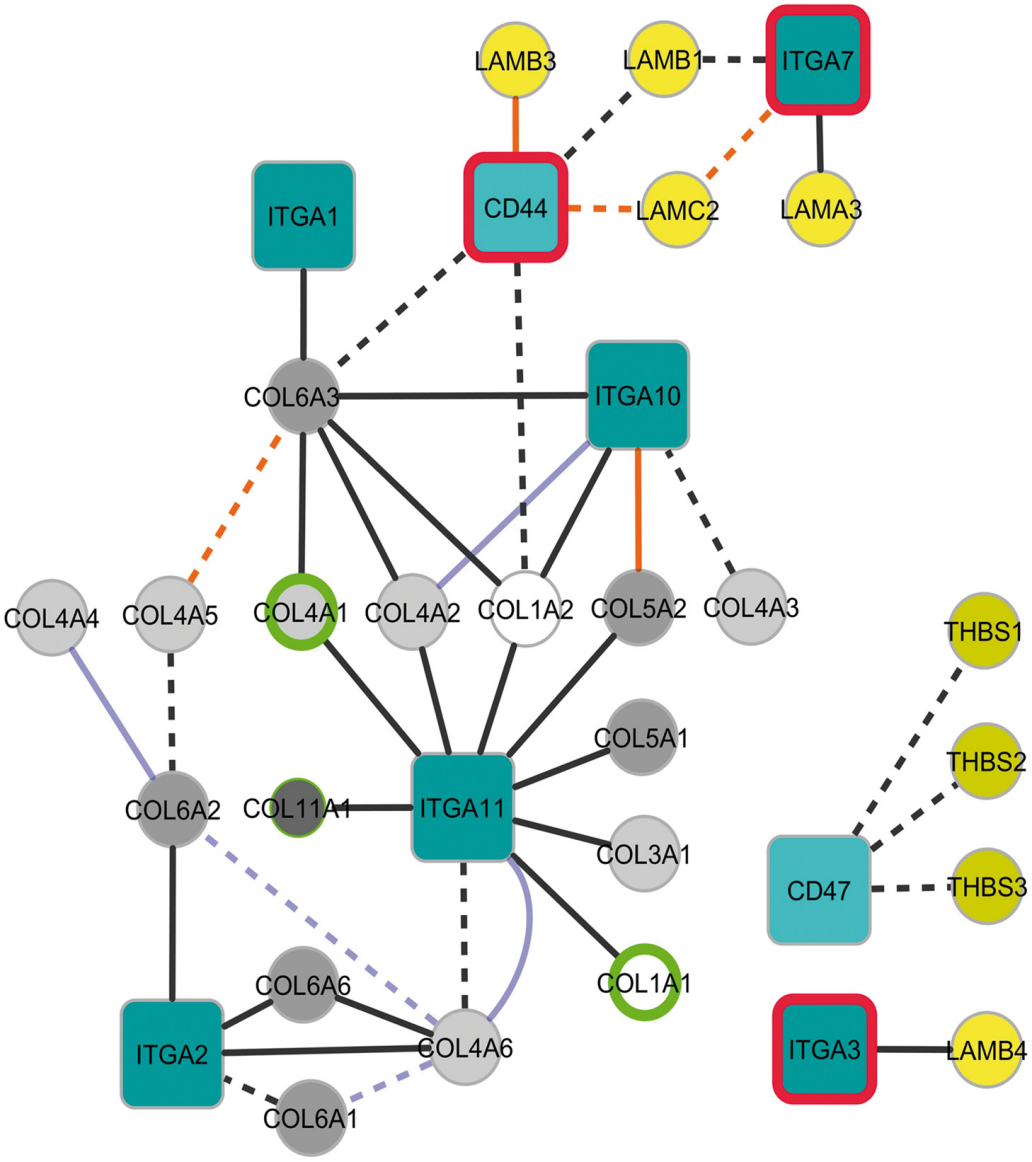
Extracellular matrix gene correlation network. This network represents Pearson correlations (R >0.8, p-value<0.005) computed considering expression values of genes of interest according to the microarray data and using Cytoscape tool. We selected those genes on the ECM-receptor interaction KEGG pathway and COL6A genes. Continuous lines represent positive correlations and discontinuous lines negative ones. Those correlations that are significant in patients´cells only are represented in black lines. Orange lines represent those correlations that are present in both patients and control cells but are of different sign (positive or negative) whereas those that have the same sign are represented by lilac lines. Red lines around gene symbols represent significantly over-expressed genes and green lines those that were under-expressed in patients´ fibroblasts relative to control fibroblasts.

In order to find out more about the possible functional consequences of the observed gene signatures from a systems biology point of view, we decided to analyse and visualize correlations between individual genes and to build networks using Cytoscape tool. This is represented in “Fig 1”. The depicted network includes only those correlations that were significant (R >0.8, p-value<0.005) between selected genes in the KEGG Pathway “ECM-receptor interaction (hsa04512)”, mainly those encoding for integrins and other cell surface receptors, laminins, collagens, tenascins and thrombospondin, and collagen VI genes that were not previously described on that pathway. Using this tool we were able to see that the connectivity between several ECM components and transmembrane receptors was altered in UCMD fibroblasts relative to control fibroblasts. For example, three of the collagen binding integrins, *ITGA2*, *ITGA10* and *ITGA11* were largely correlated with genes for collagen I, III, IV, V, VI and XI chains in the case of disease samples only (black lines, “Fig 1”). In particular, new positive correlations appeared between collagen VI chains and cell receptors (*ITGA2* and *ITGA10*) as well as with other collagens (type I and IV). Some connections between *ITGA3*, *ITGA7*, *CD44 and CD47* genes were also modified. Moreover, some correlations although found in both patients and controls changed their sign (orange lines). For example in control cells *ITGA7* positively correlated with *LAMC2* (encoding for laminin-γ2 chain) whereas in patient cells this correlation was reversed. These findings suggest that collagen VI defects result in a major reorganization of the ECM and its interaction with cells.

**Fig 2:**
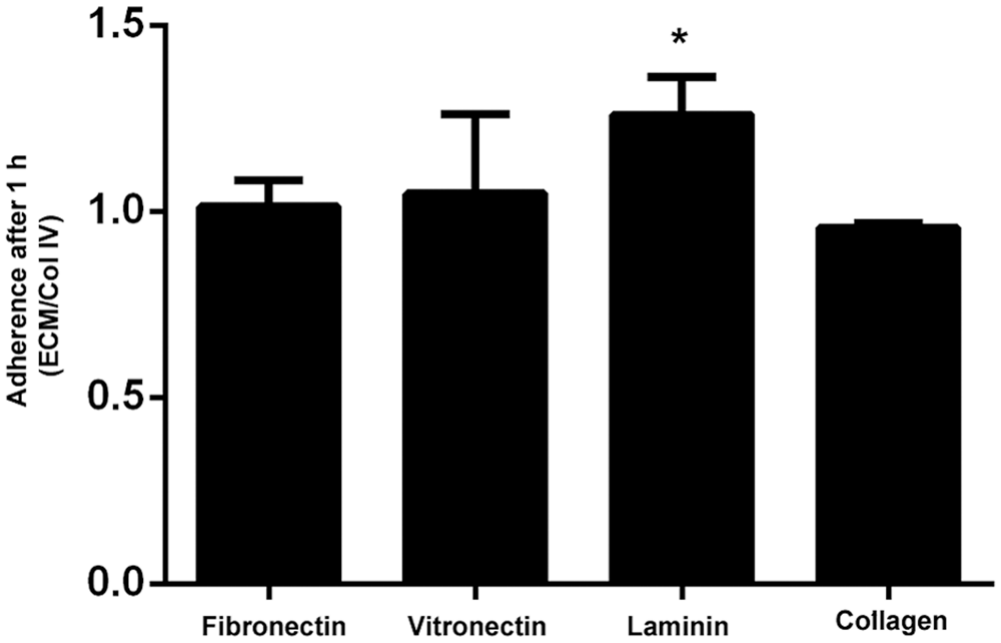
Cell adhesion assay. Adherence of UCMD fibroblasts (n = 7) relative to control fibroblasts (n = 7) for fibronectin, vitronectin, laminin and collagen type I (experiments were performed in triplicate). To normalize adhesion values between experiments, we expressed the results as a ratio between the absorbance values for collagen type IV (which was the substrate that showed the smallest variability between individual cultures and experiments, data not shown) and each ECM protein, (student t-test * p < 0.05).

To investigate this further we studied the capacity of UCMD fibroblasts (n = 7 which included the 6 fibroblasts cell lines analysed in the microarrays and an additional UCMD fibroblast sample with collagen VI deficiency) to adhere to various ECM substrates. We found that the adherence profile of UCMD cells was similar to controls for vitronectin, fibronectin and collagen type I. However, adherence to laminin was significantly increased (“Fig 2”). Any of the three integrin alpha chains that we found up-regulated in the microarray (ITGA3, ITGA6 and ITGA7) could be involved in the observed enhanced adhesion to laminin. Given that integrin-α3 has been shown to mediate fibrosis in lung and skin [27] we decided to look in more detail at the expression of this integrin in patients and control fibroblast. Here we included two additional samples from patients with mutations in collagen VI genes and the milder Bethlem myopathy (BM).

**Fig 3:**
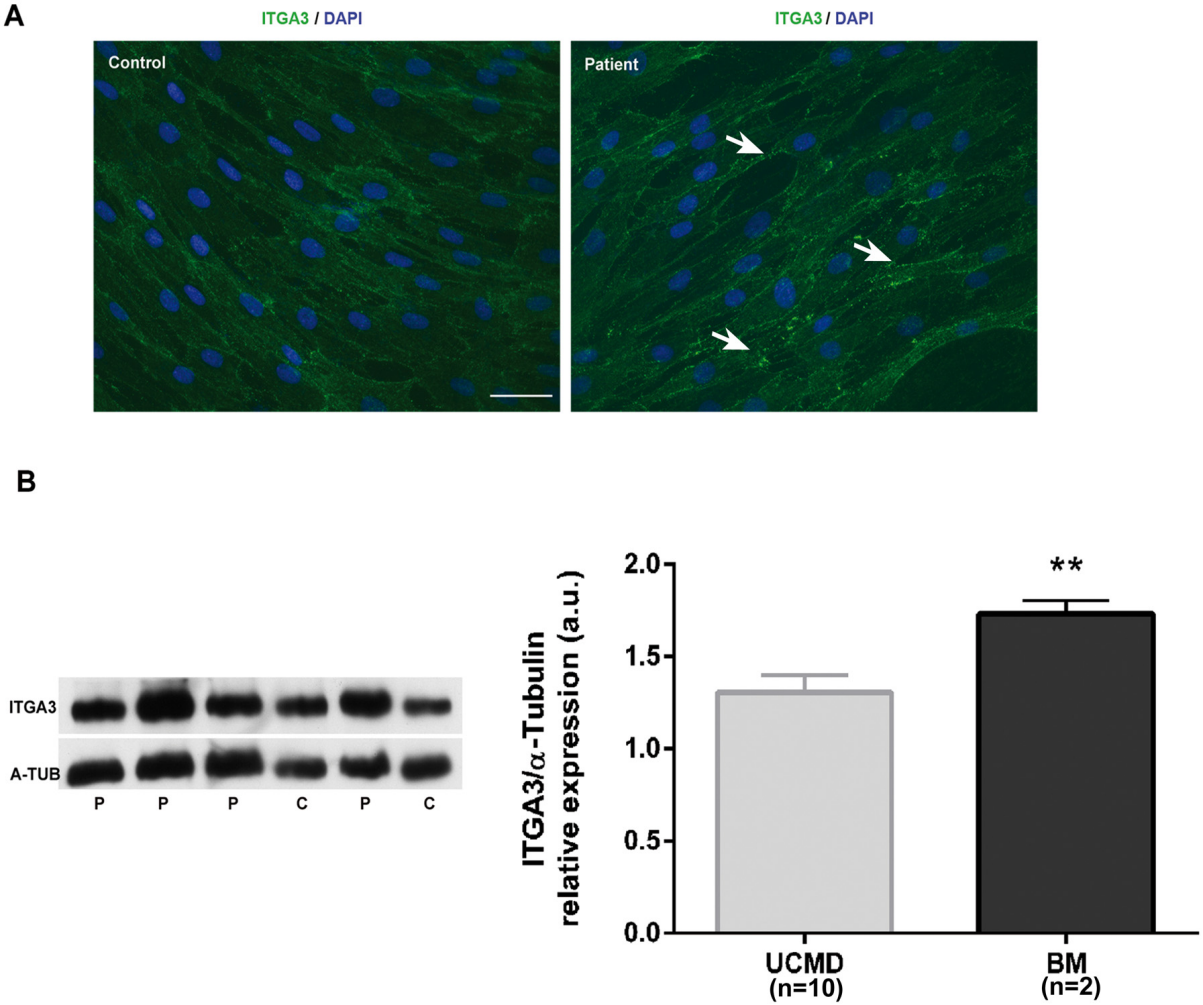
Integrin-α3 expression in collagen VI deficient fibroblasts. A. Immunofluorescence for integrin-α3 in confluent fibroblast cultures. B. Representative western blot analysis. The intensity of the bands corresponding to integrin-α3 (ITGA3) was quantified by densitometry using α-tubulin (A-TUB) as a loading control and expressed as arbitrary units relative to the control samples.

In control fibroblasts we observed a uniform punctuate labeling all over the cell surface. In patients cells the labeling appeared more intense than in control cells and in addition we observed sites of increased staining in areas of contact between two cells (“Fig 3A”). Western blot analysis was carried out from protein extracts from control (n = 2), UCMD (n = 10) and BM (n = 2) fibroblasts. A band of the expected molecular weight (119kDa) was seen in all cases. The intensity of this band was quantified by densitometry relative to α-tubulin as a loading control. On average Integrin-α3 was moderately increased in UCMD (1.3 fold) and BM extracts (1.7 fold) although it only reached statistical significance in BM patients (student t-test, p = 0.006) (“Fig 3B”).

### Regulation of gene expression: miRNAs

In order to identify key molecules potentially responsible for the observed changes in gene expression we performed Ingenuity Pathways (IPA) interaction network analysis.

**Fig 4:**
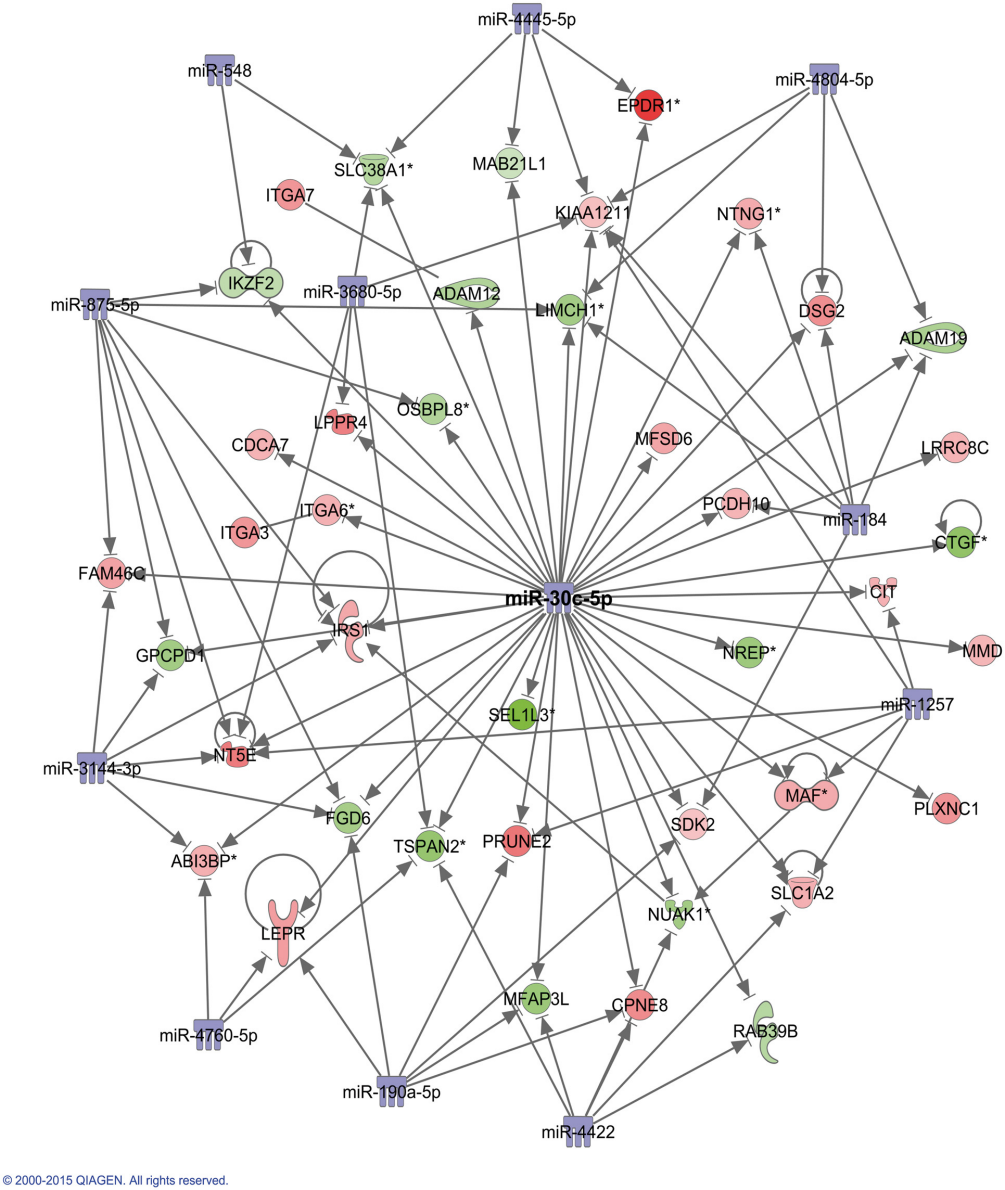
Ingenuity Pathway Analysis. Graphic representation of the network “cell cycle, skeletal and muscular system development”. Nodes represent genes and lines show the relationship between genes. The intensity of the node color indicates the degree of the up-regulation (red) or down-regulation (green) of significant genes in the P-C comparison. Non-color nodes are added by the tool. For a detailed legend refer to http://ingenuity.force.com/ipa/articles/Feature_Description/Legend.

A well scored network of genes involved in cell cycle, skeletal and muscular system development (“Fig 4”) was particularly interesting because 12 miRNAs were added by the tool, interacting with the significantly altered genes of the P-C comparison. The striking central “hub” (highly connected) of the network was miR-30c. This mRNA has been previously detected in fibroblasts where it is implicated in fibrosis since it is a powerful negative regulator of CTGF (Connective tissue growth factor) expression [28] which was down-regulated in our array (FC -2.29). Another well scored network of genes involved in protein synthesis and fibrosis was also identified (data not shown) incorporated 4 “hubs” miRNAs including miR-181a which is expressed in skeletal muscle [29].

**Fig 5:**
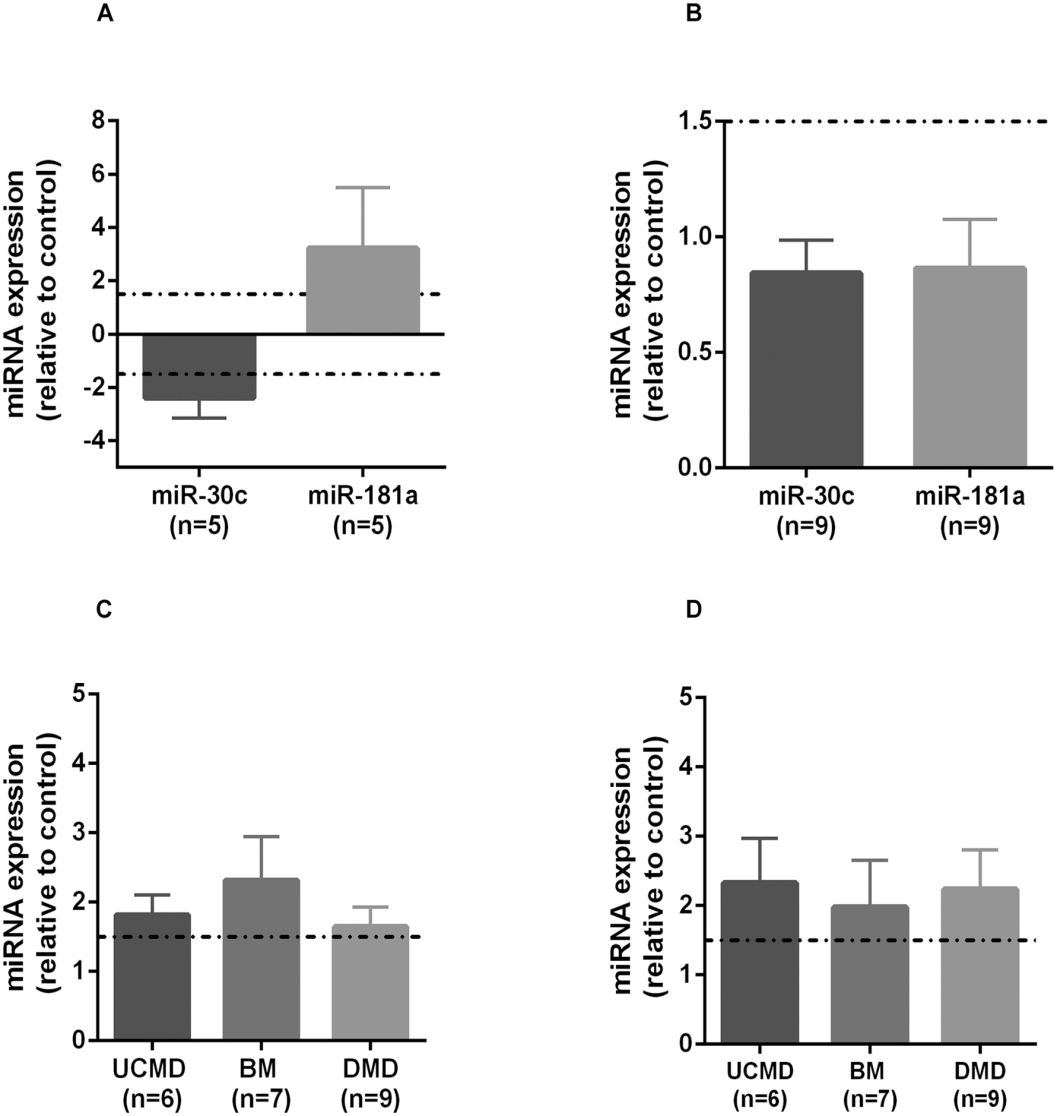
miRNAs expression analysis. Real-time PCR was used to measure relative expression of miR-181a and miR-30c in skeletal muscle (A) and fibroblasts (B) from UCMD patients and in serum samples from UCMD, BM and DMD patients for miR-181a (C) and miR-30c (D). miRNA expression level was normalized against U6 miRNA. Results were calculated relative to control samples and are represented as mean and standard error.

Besides their role as regulators of gene expression, miRNAs represent useful clinical biomarkers of different diseases including muscular dystrophies. By means of qRT-PCR we quantified miR-181a and miR-30c in UCMD muscle (n = 5) and fibroblasts (n = 9). We found that miR-181a was over-expressed in UCMD muscle relative to healthy muscle whereas miR-30c was under-expressed (“Fig 5A”). Levels of both miRNAs were decreased in RNA from UCMD skin fibroblasts (“Fig 5B”) although they did not reach the -1.5 threshold.

We also measured miR-181a and miR-30c in serum samples from UCMD (n = 6), BM (n = 7) and DMD (n = 9) patients relative to healthy controls (either children or adult). Both miRNAs were significantly elevated in all patient groups (“Fig 5C and 5D”).

Next, we calculated correlations between the levels in serum, fibroblasts and muscle of miR-181a and miR-30c in UCMD patients (expressed as deltaCT) with various histopathological parameters measured in their corresponding muscle biopsies [18] as well as with the levels of adiponectin and leptin in their serum, which are related to adiposity and metabolism. We selected those correlations achieving R value above 0.7 and p value below 0.05 (Table 6). A significant negative correlation was established between levels of miR-30c in serum and circulating adiponectin levels. At the histopathological level we found that miR-30c in UCMD muscle negatively correlated with fibre size and positively correlated with the % of pax7 positive myonuclei (an indication of the satellite cell population). UCMD miRNA_180 serum levels presented a strong negative correlation to the extent of fibrosis (measured as the % area occupied by collagen, [24]) and a positive correlation with circulating leptin levels.

**Table 6:** Correlation analysis between miR-30c and miR-181a levels and for various pathological and biochemical parameters.

Variable	Variable	p	R
miR-181a _serum	Leptin_serum	0.03892198	0.73211393
miR-181a_serum	% fibrosis_ Skm	0.01730787	-0.79922952
FiberSize_Skm	miR-30c_Skm	0.04966404	-0.70745107
Mean_fiberdiam_Skm	% adipose_Skm	0.0287597	0.75963608
Min_fiberdiam_Skm	Leptin_serum	0.01555091	-0.80665245
Min_fiberdiam_Skm	miR-30c_Skm	0.04472653	-0.718339
%MHCe_Skm	%MHCn_Skm	0.00713975	0.85265406
%MHCe_Skm	%fibrosis_ Skm	0.01877006	0.79340433
%MHCn_Skm	%fibrosis_ Skm	0.02623684	0.7673713
%Pax7_Skm	miR-30c_Skm	0.01820804	0.79560857
%Pax7_Skm	%fibrosis_ Skm	0.03621514	-0.73896665
Adiponectin_serum	miR-30c _serum	0.00277097	-0.89366847
Leptin_serum	miR-30c_fibroblasts	0.04631627	0.71475759

R: Pearson correlation coefficient; % fibrosis: percentage area occupied by collagen; %adipose: percentage area occupied by adipocytes; Skm: skeletal muscle biopsy; fiberdiam: fiber diameter; min: minimum; %MHCe:percentage of fibers immunoreactive for the embryonic isoform of myosin heavy chain; %MHCn:percentage of fibers immunoreactive for the neonatal isoform of myosin heavy chain; %Pax7:percentage of myonuclei positive for Pax7 immunolabelling.

### Effect of Ascorbic Acid (AA)

Although gene expression analysis in UCMD skin fibroblasts has not been reported before there is a previous study on the effect of AA on gene expression in healthy human skin fibroblasts [17]. We found that 104 out of the 294 genes identified by Duarte et al. were also altered in our analysis (C_AA_-C comparison).

In our study treatment of skin fibroblasts with ascorbic acid induced changes in gene expression both in UCMD and control cells. However, the number of genes whose expression significantly changed in response to AA in UCMD fibroblasts was almost half than in controls. When we compared the list of genes in both cases (PAA-P and CAA-C comparisons) we found that 307 genes were common for UCMD and control cells, 267 genes were unique for the UCMD group and 777 were unique for the control group. In order to ascertain how UCMD and control cells respond differently to AA we performed gene enrichment analysis using DAVID and looked *at the GO* terms that only changed in either control cells or in UCMD cells (36 and 35 GO categories respectively). Further grouping of those gene ontologies revealed that genes that changed after AA treatment in control cells only were mainly related to regulation of wound healing and angiogenesis, steroid and estrogens metabolism and response to vitamin and vitamin biosynthesis. In contrast those that changed in UCMD cells only were related to the cell-cycle and cell division and extracellular matrix organisation.

**Fig 6:**
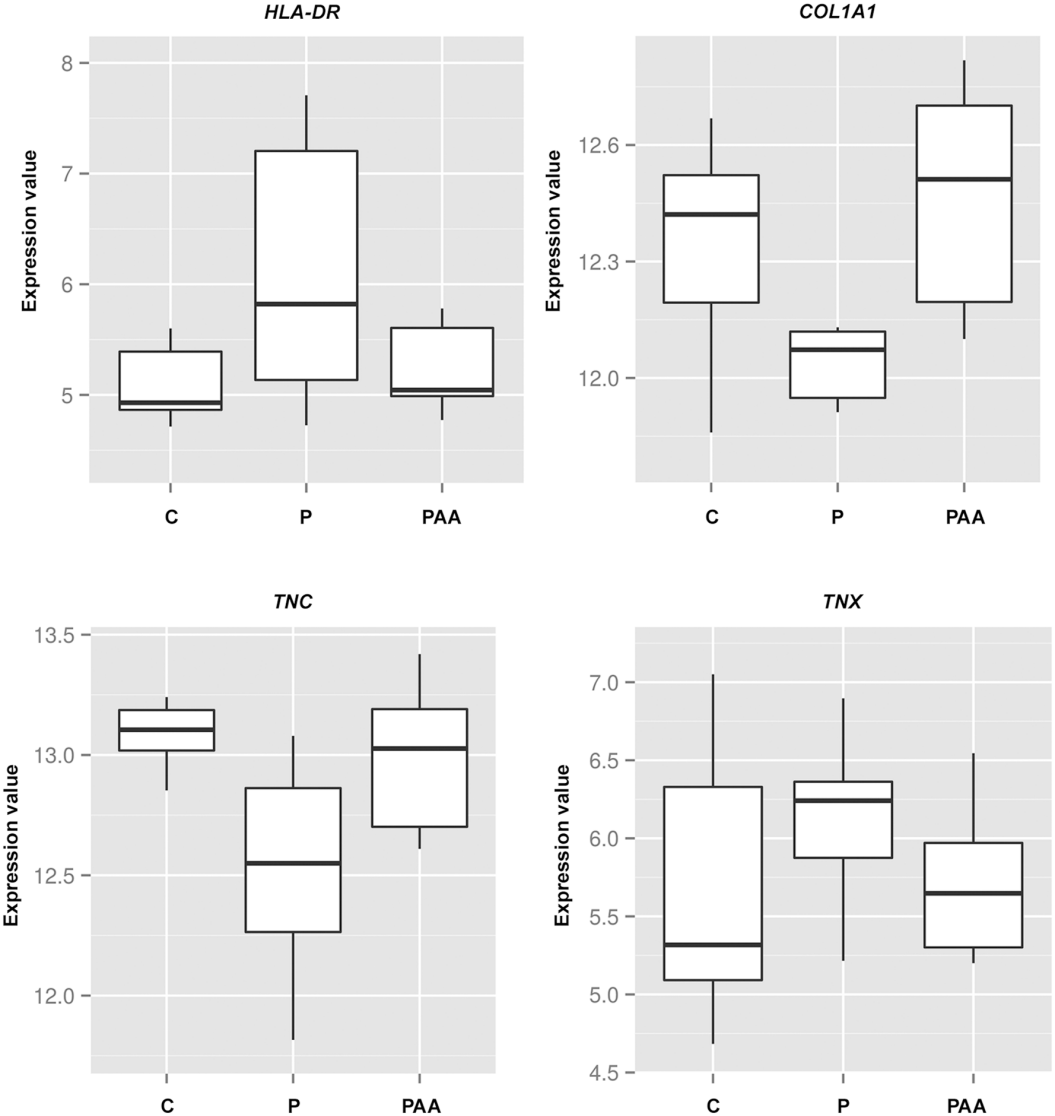
Co-variance analysis was used to plot the expression values of a selection of genes of interest in untreated patient cells (P), control cells (C) and patient cells treated with AA (P_AA_). Median and ranges are represented. A. HLA-DRA, B. COL1A1, C. TNC, D. TNX.

Given that AA treatment was able to induce important changes in gene expression we aimed to identify those genes that reversed their expression by applying AA in UCMD fibroblasts (PAA-P) with respect to those that changed purely by the effect of collagen VI mutations (P-C). We identified a set of only 40 genes that reverted sign (from down-regulated to up-regulated and vice versa) (Table 7) upon AA treatment in UCMD cells. Importantly, AA was able to change the expression of those genes to levels comparable to those found in untreated C cells. That relatively short list of genes included significantly enriched functional terms such as extracellular matrix organization and tissue regeneration. Using covariance analysis we found that PAA samples (patients cells treated with AA) grouped more closely to control cells (C) than untreated patient cells (P) cells indicating that AA acid can modify gene expression towards the basal (untreated C cells) phenotype for some genes. An example for some of those genes is shown in “Fig 6”.

**Table 7:** Genes that revert their expression in patients cells after AA treatment.

Gene	FC P_AA_—P	FC P -C
HLA-DRA	-2.0799	2.3098
KRTA P1-5	-2.1133	2.1484
EVI2A	-1.3430	2.1085
TNFRSF11B *	-1.4997	2.0947
KRT19	-1.3663	1.9729
DUSP1	-1.3470	1.9561
SEMA3B	-1.4063	1.8835
GRIA1	-2.3154	1.8560
HMGA1	-1.3774	1.7728
FOS	-1.7687	1.7060
*TIMP3	-1.3988	1.6497
SLC47A1	-1.3180	1.6351
GFRA1	-1.5015	1.6309
FXYD5	-1.4225	1.5697
*TNXB	-1.5475	1.7728
TRIL	2.2708	-2.2650
*ELN	1.6928	-2.1487
LCTL	1.3947	-2.1322
*TNC	1.4138	-2.0623
C1QTNF3	2.2280	-2.0488
OGN	2.3625	-2.0416
ARHGAP28	1.5783	-1.9912
C5orf13	1.4576	-1.9893
*COL1A1	1.6571	-1.9391
LRRC15	1.7660	-1.8854
*DPT	2.1617	-1.8734
FGD6	1.4797	-1.8577
GNB4	1.6055	-1.8471
ANGPTL1	1.5504	-1.8420
ARRDC4	1.4529	-1.8416
CSRP2	1.4479	-1.8146
*ADAM19	1.3157	-1.7809
COL10A1	2.4221	-1.7746
ACVR2A	1.5374	-1.7553
PDGFD	1.7902	-1.7188
VCAN	1.4575	-1.6998
IGF1	1.5920	-1.6075
NPNT	1.5610	-1.5337
IGSF10	1.4776	-1.4192

*Genes associated with the ECM (GO:0043062~extracellular structure organization)

## Discussion

Fibroblasts are the main source of collagen VI in skin and muscle, two of the main tissues affected in patients with mutations in COL6A genes, and therefore are the primary disease cell type. This is the first report of global gene expression profiling of collagen VI deficient dermal fibroblast. We have shown that collagen VI mutations, with almost 600 genes differentially regulated, affect several aspects of fibroblasts biology, mainly:

1. Their interaction with the surrounding environment via changes in the expression of membrane receptors and ECM components and their relationships. This would result in modified collagen fibril formation and adhesion which in turn regulates multiple signaling pathways.
2. Changes in the expression of genes involved in coagulation, inflammation and angiogenesis which are key stages of wound healing.
3. Gene expression regulation involving vitaminC and miRNAs.

### ECM organization and ECM-cell interaction

#### Collagen fibrillogenesis

The connective tissue features of collagen VI deficiency are independent of the skeletal muscle dysfunction and are directly related to the role of collagen VI in the organization and maintenance of the extracellular matrix in the affected tissues (skin, tendon, ligaments). Ultrastructural abnormalities in collagen fibrils have been reported in collagen VI deficient human and both wounded and unwounded mouse skin [30], [31], [3]and in the tendons of col6a3 deficient mice [32]. Therefore, some of the changes that we describe here may also be taking place in the tendon ECM.

For example, tenascin C in adult tendon concentrates at the myotendinous and ostetendinous junctions, its gene expression is regulated by mechanical loading and some *TNC* polymorphisms have been associated with risk of Achilles tendinopathies [33]. It would be worth investigating if the *TNC* gene is also down-regulated in tenocytes. In contrast *TNX* mRNA was over-expressed in UCMD fibroblasts as previously reported by us and others in UCMD muscle [34], [24]. Collagen VI and tenascin- X are both necessary for correct collagen fibril formation *in vitro* [35]. Specifically, tenascin X regulates the quantity of collagen fibrils whereas collagen VI regulates the rate at which those are formed. Thus, an imbalance in collagen VI and tenascin X expression levels may be contributing to impaired collagen fibrillogenesis and in changes in tendon structure and function. Thus, our data may help towards understanding the development of contractures which cause considerable disability.

Furthermore, bioinformatics analysis of gene expression data using correlation networks predicted a modified disease ECM scenario which is likely to contribute to the ultrastructural and functional changes seen in skin but could maybe also be extrapolated to muscle.

#### Cell adhesion and integrins

Cell adhesion and migration are fundamental for the formation and maintenance of tissues and underlie many processes such as morphogenesis and wound healing. High affinity adhesion is predominantly provided by integrins and CD44 [36]. We have demonstrated that adhesion of UCMD fibroblasts to laminin is increased and that the main laminin-binding integrins are over-expressed. Although integrin α3 is mainly regarded as a laminin receptor there are several reports of its binding to collagens [37]. In pulmonary fibrosis, a model of aberrant wound healing, integrin-α3 induces fibrosis via E-cadherin and TGF-B receptor [27]. Over-expression of this integrin in UCMD cells may predispose them to acquire a fibrotic profile. Integrin alpha 3 may thus provide a therapeutic target for attenuation of fibrosis without the downside effects of using non-specific TGF-B blockers. Over-expression of integrin-α7 has been described in dystrophin deficiency but not in the context of collagen VI deficiency [38]. Although it is mainly expressed in muscle cells it can be induced in other cell types including fibroblasts [39]. We did not find statistically significant changes in the expression of any of the 4 classical collagen binding integrin alpha subunits (α1, α2, α10 and α11).

Integrin mediated cell signaling modulates many aspects of cell behaviour including cell cycle, apoptosis, cell size, polarity and motility, gene expression and the response to growth factors and their activity as it is the case for TGF-B [40]. In a recent report Accorsi et al. reported up-regulation of integrin α5 and αV in muscle from laminin-α2 deficient mice [41]. Thus, integrin dysregulation and extracellular remodeling appear to be common features of collagen VI and laminin-2 deficient CMD contributing to the fibrotic and inflammatory pathology observed in both diseases. Our previous gene expression studies support the notion that both forms of CMD share common physiopathological mechanisms [24].

### Wound healing

Wound healing is an intricate process and is likely that the disarranged pattern of gene expression that we describe here disrupts its time course and final outcome resulting in abnormal scaring. For example, components of the complement cascade (C3 and C5) can directly accelerate wound healing by increasing inflammatory and fibroblast cell recruitment, collagen deposition and organization and wound strength [8]. The changes in expression of genes involved in angiogenesis and inflammation may also help explain other features seen un UCMD patients such as malar rash which is a form of erythema (redness of the skin) that appears over the cheeks and nose which is related to inflammation and increased vasodilation of superficial capillaries and is also observed in Lupus erythematosus patients.

### Role of miRNAs

Physical interaction network analysis revealed that miR-30c and miR-181a may have an important role in the regulation of gene expression in the context of UCMD. Both miRNAs were significantly increased in serum from all patients studied and correlated with different pathological and biochemical variables such as fibrosis. Therefore they could represent novel surrogate markers of muscular dystrophy in addition to the already described distromirs [42,43]. Furthermore, they could also provide novel targets to ameliorate pathology. For example, expression of miR-30c is repressed by miR-29b which in turn removes repression of connective tissue growth factor (CTGF) contributing to fibrosis [28].

### Differential effect of AA

Vitamin C (ascorbic acid) is important for the maintenance of healthy skin because of its action as antioxidant and on collagen production at the transcriptional and post-translational levels [44]. We have shown that Vitamin C treatment has different effects in healthy and collagen VI deficient fibroblasts and this could underlie some of the skin features seen in patients. The molecular basis for this difference warrants further investigation. Vitamin C (ascorbate or ascorbic acid) enters the cell via simple diffusion and active transport. The main transporters are Sodium-dependent-vitamin C transporters SVCT1 and 2 (encoded by the *SLC23A1* and *SLC23*A2 genes) and the glucose transporters Glut-1 and Glut-3 (encoded by the *SLC2A1* and *SLC2*A3 genes). We did not find significant changes in the expression of any of those 4 genes in patient cells relative to control cells. Interestingly, the expression of a set of altered functionally relevant genes was reverted towards a normal level after ascorbic acid addition, which suggests that vitamin C may have beneficial effects on some of the aspects of UCMD pathology although this needs further investigation and demonstration in vivo. Clinically, vitamin C supplementation improves wound healing in patients with pressure ulcers and Vitamin C pre-treatment accelerates wound closure in wound mouse models [45], [46]. Vitamin C is used in the treatment of mitochondrial myopathies together with other vitamins and co-factors [47] and its use has been assessed in Charcot-Marie-Tooth disease type 1 A (CMT1A) patients where it was found to be safe but without clinical benefit [48], [49].

## Conclusions

In summary, in this study we followed a systems biology approach, a global research strategy able to integrate multi-level data and thus to address the challenge of understanding rare disorders [50]. The differences observed between disease and healthy cells, not only at the gene expression level, but also at the functional, gene-gene connectivity and transcriptional regulation levels, allow us to propose a model for a “collagen VI myopathy-ECM pathway” which may serve as a starting-point for a global description of the disease and novel therapeutic options, to be explored on a larger set of patients.

## Supporting Information

S1 Table List of differentially expressed genes for each comparison. (XLSX)

S2 Table Top-ten down- and up-regulated genes. (DOCX)

S3 Table Validation of microarray results by qRT-PCR. (XLSX)

S4 Table List of enriched Gene Ontology terms (GO_BPs) for each comparison. (XLSX)
