# Supplementary material for: Transcriptome Analysis of Ullrich Congenital Muscular Dystrophy Fibroblasts Reveals a Disease Extracellular Matrix Signature and Key Molecular Regulators
Source: PLoS One. 2015 Dec 15;10(12):e0145107. doi: 10.1371/journal.pone.0145107 (PMC4686057; doi:10.1371/journal.pone.0145107)
Supplement: S2 Table — (DOCX) [file pone.0145107.s002.docx]

| \| ***Supp. Table 2.***   1. *Top-ten of down- and up-regulated genes in the comparison P_AA_-P.* \| \| \| \| \| --- \| --- \| --- \| --- \| \| ***Gene symbol*** \| ***Gene name*** \| ***FC*** \| ***FDR*** \| \| *PSAT1* \| *phosphoserine aminotransferase 1* \| *-4.44* \| *0* \| \| *ASNS* \| *asparagine synthetase (glutamine-hydrolyzing)* \| *-3.23* \| *0* \| \| *SLC7A11* \| *solute carrier family 7 (cationic amino acid transporter, y+ system), member 11* \| *-3.17* \| *0* \| \| *CBS* \| *cystathionine-beta-synthase* \| *-3.13* \| *0* \| \| *SEL1L3* \| *sel-1 suppressor of lin-12-like 3 (C. elegans)* \| *-3.13* \| *0* \| \| *TRIB3* \| *tribbles homolog 3 (Drosophila)* \| *-2.81* \| *0* \| \| *PHGDH* \| *phosphoglycerate dehydrogenase* \| *-2.75* \| *0* \| \| *PCK2* \| *phosphoenolpyruvate carboxykinase 2 (mitochondrial)* \| *-2.72* \| *0* \| \| *DDIT4* \| *DNA-damage-inducible transcript 4* \| *-2.63* \| *0* \| \| *GRIA1* \| *glutamate receptor, ionotropic, AMPA 1* \| *-2.32* \| *0* \| \| *GPR1* \| *G protein-coupled receptor 1* \| *-2.31* \| *0* \| \| *DIO2* \| *deiodinase, iodothyronine, type II* \| *4.48* \| *0* \| \| *THBS4* \| *thrombospondin 4* \| *2.84* \| *0* \| \| *WFDC1* \| *WAP four-disulfide core domain 1* \| *2.58* \| *0* \| \| *COL10A1* \| *collagen, type X, alpha 1* \| *2.42* \| *0* \| \| *RSPO3* \| *R-spondin 3* \| *2.36* \| *0* \| \| *OGN* \| *osteoglycin* \| *2.36* \| *0* \| \| *FMO1* \| *flavin containing monooxygenase 1* \| *2.30* \| *0* \| \| *SLC40A1* \| *solute carrier family 40 (iron-regulated transporter), member 1* \| *2.28* \| *0* \| \| *C1QTNF3* \| *C1q and tumor necrosis factor related protein 3* \| *2.28* \| *0* \| \| *TRIL* \| *TLR4 interactor with leucine-rich repeats* \| *2.27* \| *0* \|   ***B.*** *Top-ten down- and up-regulated genes in the comparison P_AA_-C_AA_*   \| ***Gene symbol*** \| ***Gene name*** \| ***FC*** \| ***FDR*** \| \| --- \| --- \| --- \| --- \| \| *DACT1* \| *dishevelled-binding antagonist of beta-catenin 1* \| *-4,58* \| *0* \| \| *PSAT1* \| *phosphoserine aminotransferase 1 asparagine synthetase (glutamine-hydrolyzing)* \| *-4,22* \| *0* \| \| *CBS* \| *cystathionine-beta-synthase* \| *-3,39* \| *0* \| \| *ASNS* \| *Principio del formulario*  *asparagine synthetase (glutamine-hydrolyzing)Final del formulario* \| *-3,35* \| *0* \| \| *PPAPDC1A* \| *Principio del formulario*  *phosphatidic acid phosphatase type 2 domain containing 1AFinal del formulario* \| *-3,28* \| *0* \| \| *GPC4* \| *glypican 4* \| *-3,19* \| *0* \| \| *SLC38A4* \| *solute carrier family 38, member 4* \| *-3,15* \| *0* \| \| *CXCR7* \| *Principio del formulario*  *chemokine (C-X-C motif) receptor 7Final del formulario* \| *-2,95* \| *0* \| \| *PHGDH* \| *phosphoglycerate dehydrogenase phosphoenolpyruvate carboxykinase 2 (mitochondrial)* \| *-2,92* \| *0* \| \| *COL11A1* \| *collagen, type XI, alpha 1* \| *-2,90* \| *0* \| \| *C10orf116* \| *adipogenesis regulatory factor (ADIRF)* \| *5,91* \| *0* \| \| *CLEC3B* \| *C-type lectin domain family 3, member B* \| *5,80* \| *0* \| \| *EPDR1* \| *ependymin related 1* \| *5,43* \| *0* \| \| *APCDD1* \| *adenomatosis polyposis coli down-regulated 1* \| *5,31* \| *0* \| \| *EMB /// EMBP1* \| *Principio del formulario*  *embiginFinal del formulario* \| *4,94* \| *0* \| \| *NTN4* \| *netrin 4* \| *4,49* \| *0* \| \| *ADH1B* \| *Principio del formulario*  *alcohol dehydrogenase 1B (class I), beta polypeptideFinal del formulario* \| *4,41* \| *0* \| \| *INHBB* \| *inhibin, beta B* \| *4,34* \| *0* \| \| *TNFRSF11B* \| *Principio del formulario*  *tumor necrosis factor receptor superfamily, member 11bFinal del formulario* \| *4,26* \| *0* \| \| *SAMD5* \| *Principio del formulario*  *sterile alpha motif domain containing 5Final del formulario* \| *4,19* \| *0* \|   ***C.*** *Top-ten of down- and up-regulated genes in the comparison C_AA_-C.* | | | |
| --- | --- | --- | --- | --- | --- | --- | --- | --- | --- | --- | --- | --- | --- | --- | --- | --- | --- | --- | --- | --- | --- | --- | --- | --- | --- | --- | --- | --- | --- | --- | --- | --- | --- | --- | --- | --- | --- | --- | --- | --- | --- | --- | --- | --- | --- | --- | --- | --- | --- | --- | --- | --- | --- | --- | --- | --- | --- | --- | --- | --- | --- | --- | --- | --- | --- | --- | --- | --- | --- | --- | --- | --- | --- | --- | --- | --- | --- | --- | --- | --- | --- | --- | --- | --- | --- | --- | --- | --- | --- | --- | --- | --- | --- | --- | --- | --- | --- | --- | --- | --- | --- | --- | --- | --- | --- | --- | --- | --- | --- | --- | --- | --- | --- | --- | --- | --- | --- | --- | --- | --- | --- | --- | --- | --- | --- | --- | --- | --- | --- | --- | --- | --- | --- | --- | --- | --- | --- | --- | --- | --- | --- | --- | --- | --- | --- | --- | --- | --- | --- | --- | --- | --- | --- | --- | --- | --- | --- | --- | --- | --- | --- | --- | --- | --- | --- | --- | --- | --- | --- | --- | --- | --- | --- | --- | --- | --- | --- | --- | --- |
|  |  | | |
| ***Gene symbol*** | **Gene name** | **FC** | **FDR** |
| *IL8* | interleukin 8 | -5.22 | 0 |
| *INHBE* | inhibin, beta E | -5.19 | 0 |
| *CXCL1* | chemokine (C-X-C motif) ligand 1 (melanoma growth stimulating activity, alpha) | -5.14 | 0 |
| *SCRG1* | stimulator of chondrogenesis 1 | -5.06 | 0 |
| *MEST* | mesoderm specific transcript | -4.54 | 0 |
| *CXCL2* | chemokine (C-X-C motif) ligand 2 | -4.35 | 0 |
| *CXCL6* | chemokine (C-X-C motif) ligand 6 | -4.29 | 0 |
| *GPR1* | G protein-coupled receptor 1 | -3.51 | 0 |
| *TPD52L1* | tumor protein D52-like 1 | -3.38 | 0 |
| *TGM2* | transglutaminase 2 | -3.34 | 0 |
| *THBS4* | thrombospondin 4 | 4.48 | 0 |
| *FABP3* | fatty acid binding protein 3, muscle and heart (mammary-derived growth inhibitor) | 4.03 | 0 |
| *COL10A1* | collagen, type X, alpha 1 | 4.03 | 0 |
| *MBP* | myelin basic protein | 3.55 | 0 |
| *FOXQ1* | forkhead box Q1 | 3.36 | 0 |
| *C1QTNF3* | C1q and tumor necrosis factor related protein 3 | 3.22 | 0 |
| *SFRP2* | secreted frizzled-related protein 2 | 3.07 | 0 |
| *COL14A1* | collagen, type XIV, alpha 1 | 3.05 | 0 |
| *MOBKL2B* | MOB kinase activator 3B | 3.02 | 0 |
| *OGN* | osteoglycin | 3.01 | 0 |
